# Primary and metastatic cardiac tumors: echocardiographic diagnosis, treatment and prognosis in a 15-years single center study

**DOI:** 10.1186/s13019-017-0672-7

**Published:** 2017-11-28

**Authors:** Natsumi Nomoto, Tomoko Tani, Toshiko Konda, Kitae Kim, Takeshi Kitai, Mitsuhiko Ota, Shuichiro Kaji, Yukihiro Imai, Yukikatsu Okada, Yutaka Furukawa

**Affiliations:** 1 Department of Clinical Technology, Kobe City Medical Center General Hospital, 2-1-1 Minatojima-Minamimachi, Chuo-ku, Kobe, 650-0047 Japan; 2 Basic Medical Science, Kobe City College of Nursing, 3-4 Gakuennishi-machi, Nishi-ku, Kobe, 651-2103 Japan; 3 Department of Cardiovascular Medicine, Kobe City Medical Center General Hospital, 2-1-1 Minatojima-Minamimachi, Chuo-ku, Kobe, 650-0047 Japan; 4 Department of Pathology, Kobe City Medical Center General Hospital, 2-1-1 Minatojima-Minamimachi, Chuo-ku, Kobe, 650-0047 Japan; 5 Department of Cardiovascular Surgery, Kobe City Medical Center General Hospital, 2-1-1 Minatojima-Minamimachi, Chuo-ku, Kobe, 650-0047 Japan

**Keywords:** Cardiac tumor, Echocardiography, Diagnosis, Prognosis

## Abstract

**Background:**

The frequency of primary cardiac tumors is rare at about 0.3% by autopsy. Our objective was to investigate the characteristics and locations of cardiac tumors and to provide a prognostic analysis in our hospital.

**Methods:**

We collected data on 95 patients with echocardiographic diagnosis or detection of cardiac tumors in a prospective analysis from 1999 to 2014. The median follow-up period was 43 months (0.5–183 months).

**Results:**

The subjects included 56 men and 39 women with a mean age of 65 years. Clinical diagnosis revealed primary tumors in 61 patients (64%) and secondary metastatic tumors in 34 patients (36%). In the 61 patients, 41 patients (67%) underwent surgery and tissue samples were obtained. Of these 41 patients, benign tumors were found in 30 cases (73%). One patient (2%) was diagnosed with thrombus. Among the benign tumors, myxoma (67%) was the most common type followed by papillary fibroelastoma (23%). The most common site was the left atrium (35%) followed by the right atrium (25%). Primary malignant tumors were diagnosed in 10 cases (24%), including 6 angiosarcomas, 3 lymphomas, and 1 leiomyosarcoma. The diagnostic accuracy of echocardiography was 80%. The patients with benign tumors were all alive at the end of the follow-up period. In contrast, 7 patients with malignant tumors died (70%) (*p* < 0.0001).

**Conclusions:**

Our data is in line with previous literature. Our study also suggests the necessity of extending our knowledge of the characteristics of cardiac tumors for diagnosis.

## Background

Cardiac tumors are classified into primary tumors that arise from part of the heart or metastatic tumors that involve the heart. Primary cardiac tumors include benign and malignant tumors. Primary cardiac tumors are rare and the incidence is from 0.001 to 0.3% by autopsy [1, 2]. There have only been a few published studies about cardiac tumors and their management in the past half century. The majority of papers about cardiac tumors report only on a limited number of primary cardiac tumors, especially benign tumors. Cardiac tumors present various clinical situations. The most common primary cardiac tumor is cardiac myxoma, and it was reported that 50–70% of all primary cardiac tumors were myxomas [2, 3].

We have been able to detect and accurately diagnose cardiac tumors with clinically useful imaging techniques, especially echocardiography. We investigated prospectively the characteristics of cardiac tumors that have been diagnosed by transthoracic echocardiography (TTE) or transesophageal echocardiography (TEE) at our hospital. We performed a comparison of the tumors with the pathological data from patients who underwent surgery. Moreover, we studied the clinical course of each case with a cardiac tumor in a single center.

## Methods

We prospectively identified 95 patients who had complete echocardiographic records and who were diagnosed or detected with cardiac tumors from August 1999 to December 2014.

We confirm that our study complied with the principles of the declaration of Helsinki. Approval to conduct this study was obtained from the Institutional Review Board in our hospital. All participants gave their informed consent to participate in the study.

The medical records of all the cardiac tumor patients were reviewed and details regarding diagnosis, treatment, and follow-up were obtained after echocardiographic examination. We calculated overall survival times from the date of surgery to the date of death or to the date when we were able to confirm the patient’s survival.

### Echocardiography

Two-dimensional transthoracic echocardiography (TTE) was performed by experienced sonographers according to a standardized echocardiographic protocol using commercially available equipment (ARTIDA/Aplio SSA-770A Toshiba, Tochigi, Japan; HDI 5000/iE33/EPIQ7c Philips Medical Systems, Andover, MA, USA; ACUSON SC2000 Siemens, Munchen, DEU). Images were obtained using standard views. Transesophageal echocardiography (TEE) was performed using commercially available equipment and standard imaging planes for patients who needed a more detailed examination. Some patients were not able to undergo TEE because of their physical condition.

### Diagnosis of tumor type

The tumor type was diagnosed based on location, site of attachment, shape, size, mobility and other morphologic characteristics on echocardiography. For the diagnosis of malignant tumors, the localization and growth of the tumor lesions, permeation to the great vessels, and the presence of malignancy characteristics were evaluated as follows; about localization: intracardiac, extracardiac,; about growth: invasion, infiltration, compression; about surface/border: smooth, filiform, rough. Other characteristics associated with malignancy were defined as pericardial effusion and direct invasion into the myocardium. Metastatic tumors were easily diagnosed if the tumors showed direct invasion in addition to the above characteristics. However, cases of metastatic tumors often underwent an echocardiographic examination for the purpose of determining whether cardiac metastasis might exist. All tumors were evaluated independently by two experienced investigators blinded to the patient’s clinical diagnosis. All pathology records were reviewed and confirmed by a single pathologist. We collected the pathological data from the medical records. As for the cases in the non-surgery group, the results of other imaging methods after echocardiography were confirmed.

### Statistical analysis

The Kaplan–Meier method was used to analyze survival, and the log-rank test was used for all survival differences. All statistical analyses were performed with the commercially available software package SPSS for Windows, version 23 (IBM Corporation, Chicago, IL, USA).

## Results

### Patient characteristics

There were 56 men and 39 women with a mean age of 65 years (age 65 ± 14 years). The median follow-up period was 43 months (0.5–183 months).

### Tumor characteristics

**Fig. 1:**
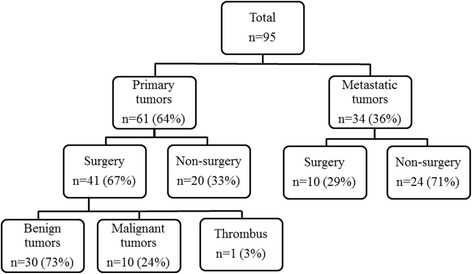
Description of the patient population

**Fig. 2:**
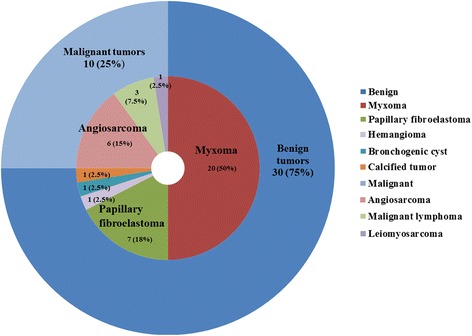
Distribution of cardiac tumors diagnosed and treated at our hospital. We excluded one case that was diagnosed as thrombus by pathological data

Of the 95 cardiac tumor cases, 61 (64%) were diagnosed as primary cardiac tumors by echocardiography. All primary cardiac tumors were diagnosed without information about the history of cardiac tumors from echocardiographic order. Thirty-four cases (36%) were detected as secondary metastatic cardiac tumors and 5 of 34 cases (15%) were accidentally diagnosed by echocardiography with no information about primary disease and other imaging data. Among the 61 patients with primary tumors, 41 patients (67%) underwent partial or complete tumor resection and had a histologic diagnosis. Twenty patients (33%) refused surgery. The majority of the surgical cases (*n* = 30, 73%) were diagnosed with benign tumors. One case was diagnosed with thrombus, not a tumor (Fig. 1). Tumors were detected in all cardiac chambers and extracardiac sites. Locations of the tumors are listed in Table 1. One case was diagnosed with thrombus, not a tumor. In 4 cases, tumors were detected at two sites in each. The most frequent tumor sites were the atrium: 35% in the left atrium and 25% in the right atrium. The tumor types are listed in Table 2. Figure 2 shows the distribution of 40 cases diagnosed pathologically by surgery. One case was excluded because of a thrombus diagnosis. The most common type of all cardiac tumors was myxoma (*n* = 20, 50%). The second-most common tumor was papillary fibroelastoma (*n* = 7, 18%). Among cases with malignant tumors (*n* = 10, 25%), angiosarcoma (*n* = 6, 15%) was the most common pathological type. Other malignant tumors were malignant lymphoma (*n* = 3, 7.5%) and leiomyosarcoma (n = 1, 2.5%).

**Table 1:** The location of cardiac tumors in 94 cases included 4 overlapping sites

Location	Total *n* = 95 (99 sites^a^)	Primary cardiac tumors *n* = 60 (61 sites)			Metastatic cardiac tumors *n* = 34 (37 sites)	
		Surgery Group *n* = 40 (41 sites)		Non-surgery Group *n* = 20 (20 sites)	Surgery Group *n* = 10 (12 sites)	Non-surgery Group *n* = 24 (25 sites)
		Benign tumor *n* = 31 (31 sites)	Malignant tumor *n* = 10 (11 sites)			
Left Atrium	34 (34%)	22 (71%)	0	5 (25%)	1 (8%)	6 (24%)
Right Atrium	25 (26%)	2 (6%)	8 (73%)	4 (20%)	4 (34%)	7 (28%)
Left Ventricle	6 (6%)	1 (3%)	0	4 (20%)	0	1 (4%)
Right Ventricle	7 (7%)	0	2 (18%)	1 (5%)	1 (8%)	3 (12%)
Aortic Valve	10 (10%)	5 (16%)	0	5 (25%)	0	0
Mitral Valve	1 (1%)	0	0	1 (5%)	0	0
Pulmonary Valve	1 (1%)	1 (3%)	0	0	0	0
Inferior vena cava	6 (6%)	0	0	0	4 (34%)	2 (8%)
Aorta	4 (4%)	0	0	0	1 (8%)	3 (12%)
Pulmonary Artery	2 (2%)	0	1 (9%)	0	0	1 (4%)
Pericardium	3 (3%)	0	0	0	1 (8%)	2 (8%)

^a^In 4 cases, tumors were detected at two sites in each

**Table 2:** Histopathology of primary cardiac tumors

Pathology	No. of patients
Benign tumors	30
Myxoma	20 (67%)
Papillary fibroelastoma	7 (23%)
Hemangioma	1 (3%)
Bronchogenic cyst	1 (3%)
Calcified tumor	1 (3%)
Malignant tumors	10
Angiosarcoma	6 (60%)
Malignant lymphoma	3 (30%)
Leiomyosarcoma	1 (10%)
Thrombus	1

As for the cases with primary cardiac tumors in the non-surgery group, benign cardiac tumors were diagnosed in all 20 cases. We diagnosed myxoma in 13 cases, papillary fibroelastoma in 6 cases and lipoma in one case. After echocardiography, we confirmed other imaging data. These cases were highly suspected primary benign tumors, but no definitive diagnosis could be obtained.

### Diagnosis of cardiac tumor

Table 3 shows the comparison of the echocardiographic diagnosis with the pathological diagnosis. By echocardiography, 41 cases were diagnosed with primary cardiac tumors. TEE was performed in 27 (66%) of the operated 41 patients. Four cases (10%) could only be diagnosed by TEE, not by TTE. Tumor types diagnosed by TEE were myxoma in 1 case and angiosarcoma in 3 cases. The patients diagnosed with papillary fibroelastoma and the patients diagnosed with angiosarcoma by echocardiography were all concordant with pathological results.

**Table 3:** The diagnostic accuracy of echocardiography

Pathology	Echocardiographic diagnosis	Accuracy
Myxoma (*n* = 20)	Myxoma (*n* = 20)	100%
Thrombus (*n* = 1)	Myxoma	0%
Papillary fibroelastoma (*n* = 7)	Papillary fibroelastoma (*n* = 7)	100%
Angiosarcoma (*n* = 6)	Angiosarcoma (*n* = 5)	83%
	No definite diagnosis (*n* = 1)	
Malignant lymphoma (*n* = 3)	No definite diagnosis	0%
Leiomyosarcoma (*n* = 1)	No definite diagnosis	0%
Bronchogenic cyst (*n* = 1)	No definite diagnosis	0%
Hemangioma (*n* = 1)	No definite diagnosis	0%
Calcified amorphous tumor (*n* = 1)	Calcified amorphous tumor	100%

**Fig. 3:**
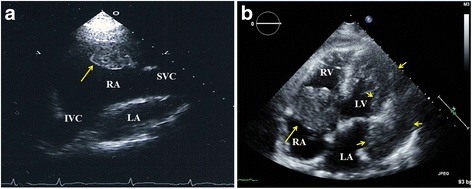
Representative cases incorrectly diagnosed by transthoracic echocardiography. **a**. Horizontal section of a right parasternal view. Thrombus attached to RA appendage (arrow). We misdiagnosedthis as an RA myxoma. **b**. Left parasternal four-chamber view. We could not diagnose the tumor type before surgery. A leiomyosarcoma arose from the coronary sinus and was detected around the LV (arrows). RA = right atrium, LA = left atrium, IVC = inferior vena cava, SVC = superior vena cava, LV = left ventricle, RV = right ventricle

Otherwise, in 6 cases with angiosarcoma diagnosed by pathology, 3 cases (50%) could not be diagnosed by TTE but were diagnosed by TEE. Two cases (33%) could be diagnosed as an angiosarcoma by TTE. One case of angiosarcoma (17%) could not have the tumor type diagnosed by echocardiography. One case was diagnosed with myxoma by echocardiography, but was found to be a thrombus on pathology. One case could not determine the tumor type by echocardiography, but was a clear hemangioma by pathology. In summary, we could not diagnose the tumor types in 7 cases (17%) by echocardiography. These tumors were malignant lymphoma in 3 patients (7.3%), and 1 case each of angiosarcoma (2.4%), leiomyosarcoma (2.4%), hemangioma (2.4%) and bronchogenic cyst (2.4%). Figure 3a shows the case with thrombus that was misdiagnosed as a right atrial myxoma by echocardiography. Figure 3b shows the case with leiomyosarcoma that could not be diagnosed by echocardiography. The diagnostic accuracy of cardiac tumors by echocardiography was 80% (33/41).

### The characteristics of secondary metastatic cardiac tumors

Thirty-four of 95 cases (36%) were detected or diagnosed as metastatic cardiac tumors by echocardiography. From the echocardiographic images, these were diagnosed as metastatic cardiac tumors for the following reasons: direct invasion to the heart, a massive pericardial effusion, or an irregular mass. We performed echocardiography in 6 cases without the information about primary disease and other imaging data from the echocardiographic order. The metastatic cardiac tumors were initially diagnosed by echocardiography in 5 of 6 cases. However, one case could not be distinguished from a pericardial cyst. The diagnostic rate was 15% of all metastatic tumors. The information about the primary disease or other imaging data from echocardiographic order was obtained in 28 cases before examination.

**Fig. 4:**
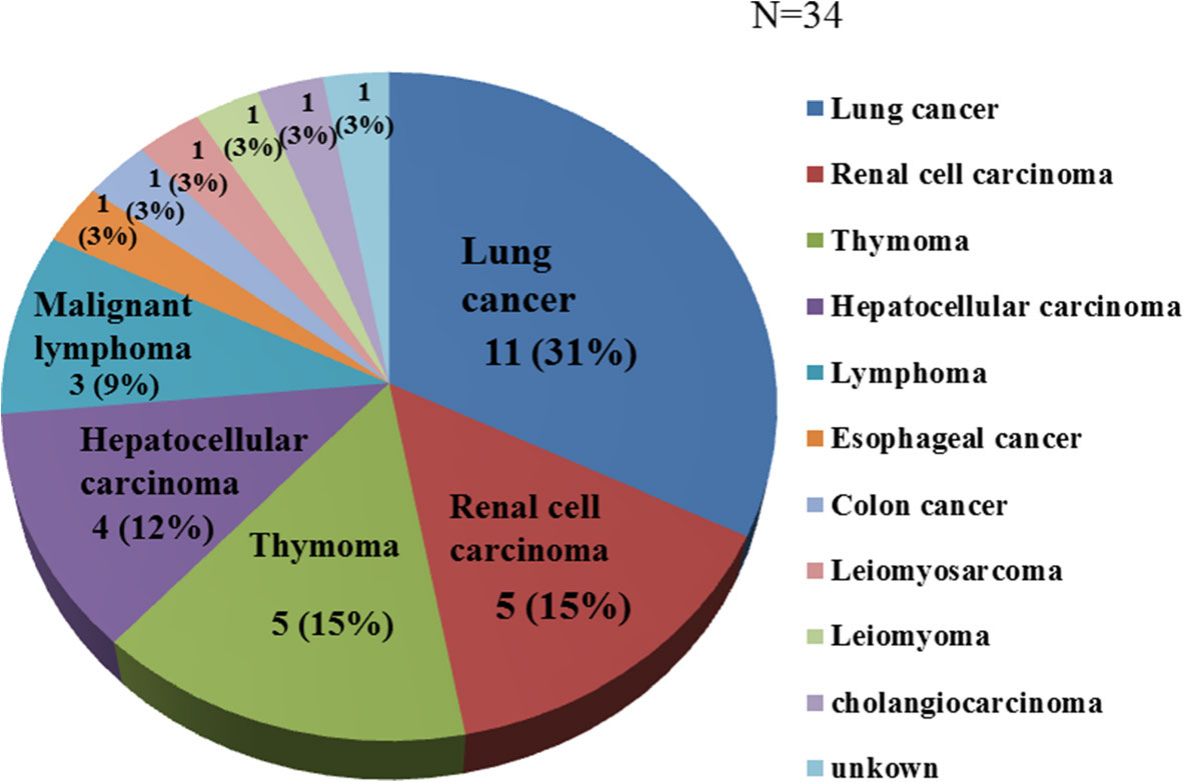
Demographics of origins of metastatic cardiac tumors

From the echocardiographic characteristics, a definite diagnose of the metastatic tumors could be made in 30 of all 34 cases, with or without information about the tumor in echocardiographic order. With echocardiographic diagnosis, a diagnosis could be made because of the specific characteristics. Concretely, the echocardiographic characteristics that allowed us to make a metastatic tumor diagnosis were direct invasion of the aorta in 4 cases, amass of inferior vena cava (IVC) in 10 cases, direct invasion of the right ventricle (RV) in 3 cases, direct invasion of the left atrium (LA) in 3 cases, direct invasion of the right atrium (RA) in 2 cases, penetration of interatrial septum in one case, tumors that continued into the left atrium from the pulmonary vein (PV) in 2 cases and a tumor that continued into the right atrium from the supra vena cava in one case. One case diagnosed as lymphoma was detected as a metastatic tumor at the tricuspid annulus, which is a specific metastatic site for lymphoma. The metastatic tumors at the pericardium and pericardial effusion found in 3 cases were diagnosed accurately because of primary disease from echocardiographic order. Otherwise, the irregular mass in the chambers could be suspected as malignant tumors in 3 cases. However, we unable to diagnose whether these were metastatic tumors or not only by echocardiography. The most frequent tumor sites were the atrium: 30% in the right atrium and 19% in the left atrium. The second-most common tumor site was the inferior vena cava (16%). Primary diseases of metastatic cardiac tumors are shown in Fig. 4. The most common primary disease was lung cancer followed by renal cell carcinoma and thymoma. Ten of the 34 cases (29%) underwent surgery for their cardiac tumors.

### Operative data

All benign tumors were completely resected without major complications. The patient with a bronchogenic cyst was implanted a permanent epicardium pacemaker for atrioventricular block.

In ten patients with primary malignant tumors, one patient underwent surgery twice for recurrence.

Two patients with sarcoma underwent complete resections.

Seven of 10 patients (70%) required reconstructions of RA with autologous or bovine pericardium. One patient required tricuspid annuloplasty and aortocoronary bypass to right coronary artery.

One patient with direct invasion of the ascending aorta underwent concomitant ascending aorta replacement.

Ten patients with secondary metastatic tumors underwent surgery. All IVC tumors were completely resected. The IVC wall was directly incised in 3 patients. In one patient, the free wall of the RA was incised and then an IVC tumor was resected. The patient with direct tumor invasion of the aorta received a replacement of the descending aorta with an artificial blood vessel. In the patient whose tumor was a continuous invasion from the PV into LA, the tumor with the partial portion of the LA wall and PV was resected. Thereafter, the posterior wall of the LA was repaired with self pericardium. In the patient whose tumor was directly invading the RA wall, the tumor with the anterior wall of the RA was resected and the defect portion was recreated with equine pericardium.

### Mortality

**Fig. 5:**
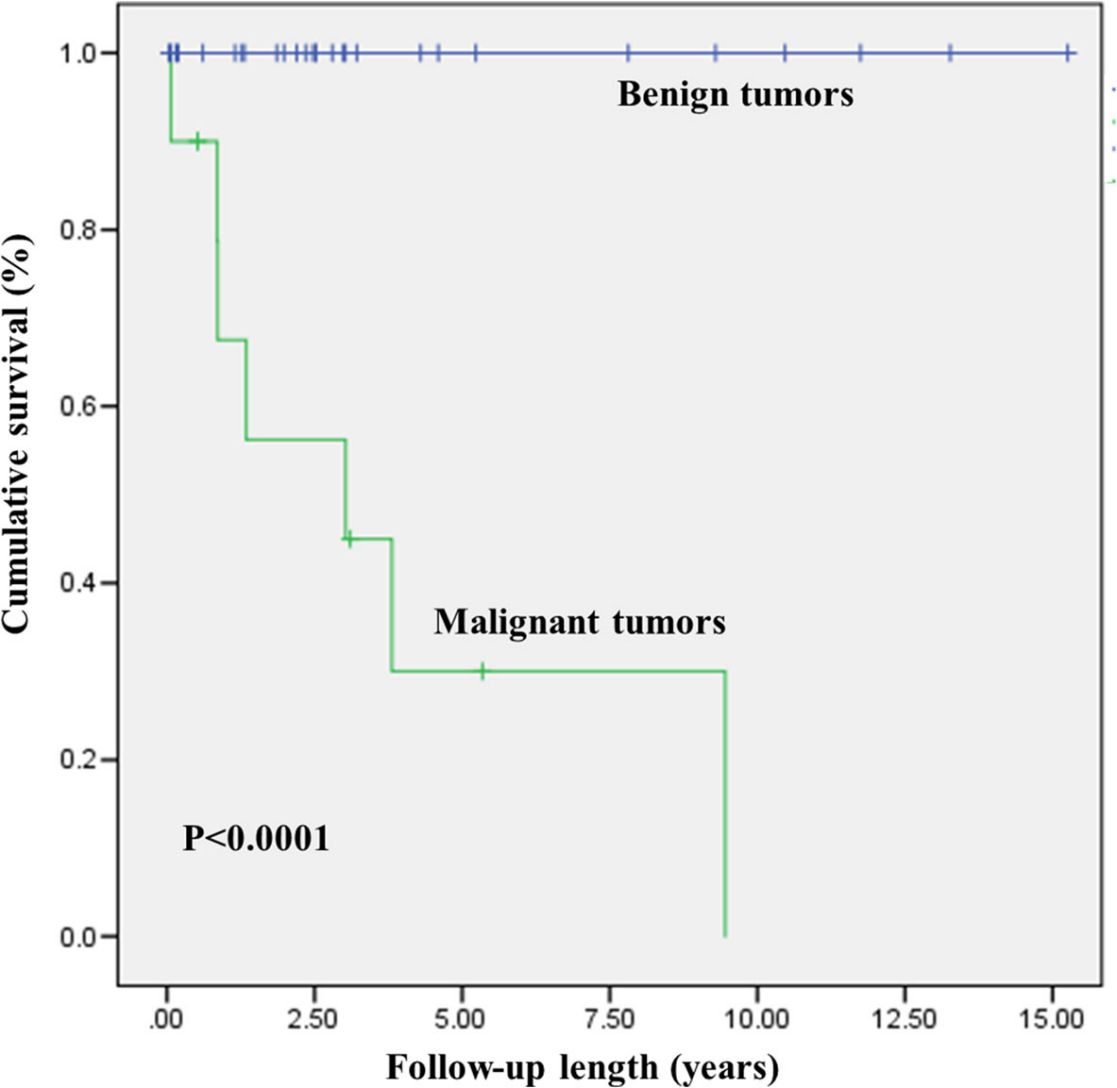
Kaplan–Meier survival curves of patients undergoing surgery for cardiac tumors. Overall survival of the patients with benign and malignant cardiac tumors

The median follow-up period was 43 months (0.5–183 months). The patients diagnosed with benign tumors were all alive at the end of the follow-up period (*p* < 0.0001; Fig. 5). The mean survival of the patients with malignant tumors was 113.5 ± 34.1 months. Seven patients with malignant tumors died (70%). Five of these 7 patients were diagnosed with angiosarcoma and the othertwo were each diagnosed with leiomyosarcoma or malignant lymphoma. All of the patients with malignant tumors received adjuvant chemotherapy after resection. The median survival for the patients with angiosarcoma was 42.2 months (10–113 months). Median survival for the patients with other malignant tumors was 21.8 months (0.8–64 months). The 1-month mortality of patients with malignant cardiac tumors was 30% (Fig. 5).

In the 20 non-surgery patients with primary tumors, a primary malignant tumor was found in only one patient. Seventeen patients were followed up. Three patients died. The patient diagnosed with angiosarcoma died 47 days after the diagnosis. The other 2 patients diagnosed with benign tumor died because of non-cardiac diseases. The maximum survival was 6-years.

In the 24 non-surgery patients with metastatic malignant tumors, 20 patients could be followed up. Eighteen patients died. The maximum survival for these 18 patients was 7-years in the patient diagnosed with lymphoma. The minimum survival was 2 days in the patient with colon cancer. The maximum survival for all patients with metastatic malignant tumors was 11.5-years.

## Discussion

Indeed, there might be few new information, however, some clinically important information has been found in our study. Firstly, our study reconfirmed previous reports about cardiac tumors. Previously, echocardiography had been shown to be a useful technique for detecting cardiac masses [4]. Secondly, the present study reconfirmed the echocardiographic characteristics of cardiac tumors and histological diagnoses. Moreover, this study also confirmed the utility of echocardiography in the diagnosis of uncommon cardiac tumors in a single center. This study had a clinical importance to address the characteristics of cardiac tumors and to reconfirm the usefulness and problems of echocardiography.

### Characteristics of cardiac tumors

Primary cardiac tumors are rare and the incidence is 0.001–0.3% by autopsy, while metastatic tumors to the heart are reported to be 20 to 40 times more common by an autopsy report [5]. The reported clinical incidence of primary cardiac tumors has varied between 0.001% -0.03% in most studies, with cardiac tumors as the cause of only 0.3%–0.85% of all open-heart surgeries [1, 6]. Approximately 75% of all primary cardiac tumors are benign, with myxoma accounting for at least half of the reported cases [1, 7, 8]. 25% of primary cardiac tumors are malignant with sarcoma accounting for a majority of the reported cases [9]. Kumar et al. reported that 184 of 188 cases (98%) had primary cardiac tumors, with 170 cases (92%) of benign cardiac tumors and 14 cases (8%) of primary malignant cardiac tumors. Only 4 of 188 cases (2%) were secondary metastatic tumors [9]. Other studies reported that only 3.3% of all cardiac tumors were metastases [10]. In our study, 64% (61/95) of all cardiac tumors were primary cardiac tumors and metastatic tumors were detected in 36% of cases (34/95). Compared with previous reports, the numbers of metastatic tumor cases in our hospital was higher. The main reason is that many cancer patients were referred to our hospital for higher-level treatment. Therefore, the proportion of metastatic cardiac tumors was higher.

Our study confirms the results from previous studies in that the majority of primary cardiac tumors are benign (≥ 75%) and most benign cardiac tumors are atrial myxomas. Although significantly less common, papillary fibroelastoma is the second-most common benign cardiac tumor. One unusual case in our study was diagnosed with a very rare bronchogenic cyst. A calcified tumor was detected in only a single case. The premortem diagnosis of primary malignant cardiac tumors is rare. In previous studies, malignant cardiac tumors were even more rare, representing 5.1–28.7% of all cardiac tumors [11]. In our study, 25% of the primary cardiac tumors (10/40) were malignant, with angiosarcoma representing 15% of all primary cardiac tumors and 60% of primary malignant tumors. The differences in the percentage of malignant cardiac tumors across studies may be explained by characteristics of the hospital. If the hospital is famous for the treatment of malignant diseases, the number of cases with primary or secondary malignant cardiac tumors may increase. Mortality in cases of primary benign tumor is low [12–14]. However, < 50% of patients with primary malignant cardiac tumors were alive by the end of the first year after diagnosis, with a sharp decrease in survival for sarcoma patients [15]. In our study, 70% of patients with primary malignant cardiac tumors were alive at the end of the first year. However, after one year, the survival rate decreased sharply. Metastatic cardiac tumors make up 0.7–3.5% of cardiac tumors. The major primary lesion was lung cancer in our study, consistent with a prior study [16].

### Echocardiographic diagnosis

Echocardiography and cardiac magnetic resonance imaging (MRI) are also useful to identify cardiac tumors and malignant masses [17]. However, TTE is now the principle imaging technique for cardiac tumor detection. It can usually provide adequate diagnostic information, such as a cardiac site, size, and shape of a tumor. Thereafter, the mass can be checked in detail by computed tomography (CT) or MRI. In our practice, we can accurately diagnose the masses that have characteristic images by echocardiography. The diagnostic accuracy of echocardiography is good (80%). The diagnosable tumors include myxoma, papillary fibroelastoma, and angiosarcoma [18, 19]. The most common tumor type is myxoma, and second is papillary fibroelastoma. These tumors can be diagnosed easily by echocardiography because of specific characters, and we think that this is the reason for the high diagnostic accuracy of echocardiography for cardiac tumors. Therefore, echocardiography is very useful for the diagnosis of benign cardiac tumors. Otherwise, it is difficult to diagnose the rarer tumors. For these cases, TEE is a useful method of imaging cardiac masses. In our study, we could not diagnose the tumor type using TTE in 4 patients, but were able to diagnose them usingTEE. Recently, 3-dimensional TEE can provide a clearer view of the characteristics of cardiac mass. However, TEE is more invasive than TTE. Therefore, the necessity of TEE must be considered individually. Further effort needs to be taken to understand the tumor characteristics on echocardiography. However, it is difficult to diagnose cases with rare types of tumors using echocardiography.

### Clinical implications

Primary cardiac tumor is very rare, however, cardiac tumors are sometimes found at the time of echocardiographic examination. Therefore, we should recognize the echocardiographic characteristics of cardiac tumors and make an effort to diagnose them accurately. As for benign tumors, the specific echocardiographic characteristics and locations are very important for diagnosis.

We canreconfirm the importance of echocardiographic characteristics.

When we suspect malignant tumors, the extent of tumor invasion must be examined attentively to decide the patient’s operative status. Furthermore, surgeons need information regarding how much of the tumor can be removed The extent of resection is an important factor contributing to the prognosis. Many cases of metastatic cardiac tumors could be diagnosed by other imaging modalities. Malignant diseases generally undergo follow-up examination, especially using computed tomography. Therefore, the importance of echocardiography may be the diagnostic tool for the progression degree, rather than the diagnosis itself. However, in fact, the small number of cases could be initially diagnosed by echocardiography. We recommend that the chief physician follow up by not only CT but also echocardiography when the patients with malignancy arefollowed up. We also must understand the limitations of echocardiography.

### Limitations

First, this study was performed in a single center. Therefore, the number of cases was very small. We need to perform a multicenter study that includes more patients with cardiac tumors. Second, we only evaluated biopsy-proved masses, and therefore, no conservatively managed masses were included. Third, the follow-up period varied by the kind of tumor, especially in primary malignant tumors. Fourth, we only investigated the diagnostic accuracy and importance of echocardiography. We also need to study the diagnostic accuracy and importance of computed tomography and magnetic resonance imaging. Finally, our data may be biased because many patients with malignant tumors visited our hospital for advanced treatment. This point can be addressed by performing a multicenter study.

## Conclusion

Cardiac tumor is a rare and important disease of the heart. Our results are consistent with previous reports in regards to the incidence, tumor characteristics and prognosis of cardiac tumors. Echocardiography offers advantages in early identification of cardiac masses noninvasively and in providing clinically important information about the characteristics of the masses. Our study also suggests the necessity of extending our knowledge of the characteristics of cardiac tumors.

## Abbreviations

CT: Computed tomography
MRI: Magnetic resonance imaging
TEE: Transesophageal echocardiography
TTE: Transthoracic echocardiography
